# A single-mode tunable plasmonic sensor based on an 8-shaped resonator for cancer cell detection

**DOI:** 10.1038/s41598-023-41193-3

**Published:** 2023-08-26

**Authors:** Mohammad Danaie, Leila Hajshahvaladi, Elham Ghaderpanah

**Affiliations:** 1 Faculty of Electrical and Computer Engineering, Semnan University, Semnan, Iran; 2 Photonics Research Laboratory, Electrical Engineering Department, Amirkabir University of Technology, Tehran, Iran

**Keywords:** Integrated optics, Optical sensors

## Abstract

In this paper, a novel 8-shaped resonator coupled to metal–insulator–metal waveguides is used for designing plasmonic filters and sensors. The resonator supports two resonance modes, which result in peaks in the transmission spectrum of the structure. A Q-factor of 247.4 which can reach up to 270 at the wavelength of 1187.5 nm is observed. By placing vertical and horizontal metal blades in the resonator, two tunable single-mode plasmonic filters are obtained at the first and second resonance modes, respectively. The effect of structural parameters on the transmission spectrum is investigated using the finite-difference time-domain (FDTD) method. Based on the obtained results, the proposed plasmonic structure can be used for biosensing applications such as the detection of basal cancer cells with a sensitivity of 1200 nm/RIU. It is of great significance that both the sensitivity and Q-factor values for the proposed structure are higher than most recent sensors reported in the literature. Therefore, the proposed structure is a potentially promising candidate for filtering and sensing applications.

## Introduction

Over the past decade, surface plasmon polaritons (SPPs) have attracted significant interest due to their unique properties, such as propagating along metal–dielectric interfaces^1^. With SPPs, optical signals can be confined and controlled at a nanoscale level, thus overcoming the traditional diffraction limit of dielectric waveguides^2,3^. Optical SPPs are considered to be a promising information carrier for ultra-high density photonic integrated circuits of the next generation^4^. Metal–insulator–metal (MIM) waveguide structures are one of the most commonly used SPP-based structures for the realization of highly integrated optical circuits. A MIM structure has the advantages of providing deep sub-wavelength confinement of light and good balance between propagation length and loss, as well as ease of integration on a microchip^5,6^. Today, many plasmonic structures have been designed for a wide range of applications, including filters^7–9^, sensors^10–18^, demultiplexers^19,20^, switches^21–23^, logic gates^24^, etc. Although these structures can be realized using other techniques such as silicon photonic technology or photonic crystals, plasmonic structures occupy a much smaller area compared to the mentioned platforms^25,26^.

Since the properties of plasmonic MIM waveguides and filters are highly dependent upon the refractive index of the insulator material, they can be utilized to measure refractive index changes of the environment^27^. Thus, plasmonic MIM structures provides significant advantages for subwavelength refractive index sensing^28^. There is a great deal of interest in optical sensors due to the wide range of applications they have. Biomedical applications are one of the most significant uses of optical sensors. In particular, these sensors may be used in biosensing applications such as the detection of cancer cells, health care applications, measurement of blood components, etc.^29–33^. Optical sensors have been designed using a variety of approaches and based on various configurations, such as plasmonic^34–38^, photonic crystal^39–42^, graphene^43–50^, optical fiber topologies^51–55^ such as fiber Bragg grating, long period grating, SMS fiber structure, fiber in-line M–Z interferometer, F–P cavity, etc. Plasmonic sensors based on MIM waveguides have drawn a lot of attention because they have a smaller footprint and stronger sensing capabilities compared with other sensing techniques. Due to their small size, simplicity of integration, and good balance between light localization and transmission loss, plasmonic sensors are a good candidate to produce highly integrated optical circuits. Plasmonic sensors offer several unique advantages such as label-free detection, high sensitivity, rapid response, real-time monitoring, strong amplification of a local electric field, spectral tunability and compliance with nanotechnology^56^. Despite these advantages, they have some limitations such as limited penetration depth, limited selectivity and limited reproducibility. The fabrication of sensors based on metallic nanostructures can be challenging. Small variations in the geometry of the structure, and changes in temperature, humidity, and pH can affect the sensor response. Notwithstanding these limitations, plasmonic sensors have found many applications in different fields. Hence, researches are being done to develop and improve the performance and capabilities of these structures by eliminating these limitations^56,57^.

In this paper, three topologies are proposed, which consist of two MIM waveguides coupled to each other by an 8-shaped resonator. First, a tunable plasmonic filter structure based on the 8-shaped resonator is investigated which supports two resonances. Then, to provide a tunable single-mode filter, the second and third structures are proposed to support the first and second resonances, respectively. The performance of the proposed structures is evaluated by the finite difference time domain (FDTD) method. Finally, the performance of the third structure as a high-sensitivity plasmonic refractive index biosensor is evaluated for detection of basal cancer cell which is responsible for causing skin cancer in human body. The resonance wavelengths of a ring resonator depend on the optical path of the ring resonator and the mode order. The optical path is defined as 2πRn_eff,_ where R is the radius, and n_eff_ is the effective refractive index. An 8-shaped resonator occupies less area compared to a circular or a ring-shaped resonator with the same circumference, which leads to a smaller footprint. Here, we first investigate the behavior of the 8-shaped resonator and observe the resonance modes and later we modify the resonator to make it single-mode. In the first structure, the overlap of two resonances may complicate spectral identification when the resonances are shifted. Hence, the first structure is not optimum for sensing applications that need reliable and accurate spectral shifts. For sensing applications, a single-mode resonance is preferred. For this reason, we have modified the 8-shaped resonator and proposed the second and third structures which exhibit a single-mode resonance. The proposed plasmonic structure can be fabricated using electron beam lithography (EBL) with adequate exposure time and focused ion beam (FIB) technique^58^. Generally, the fabrication of plasmonic structures can be costly and time-consuming, which can limit their use in some specific applications^56^.

The current paper is organized as follows: in “The proposed structure and analysis method”, the proposed structure is described and the analysis method is theoretically presented In “Results and discussion”, the simulation results are discussed. Finally, in “Biosensing application”, we will have a conclusion.

## The proposed structure and analysis method

**Figure 1:**
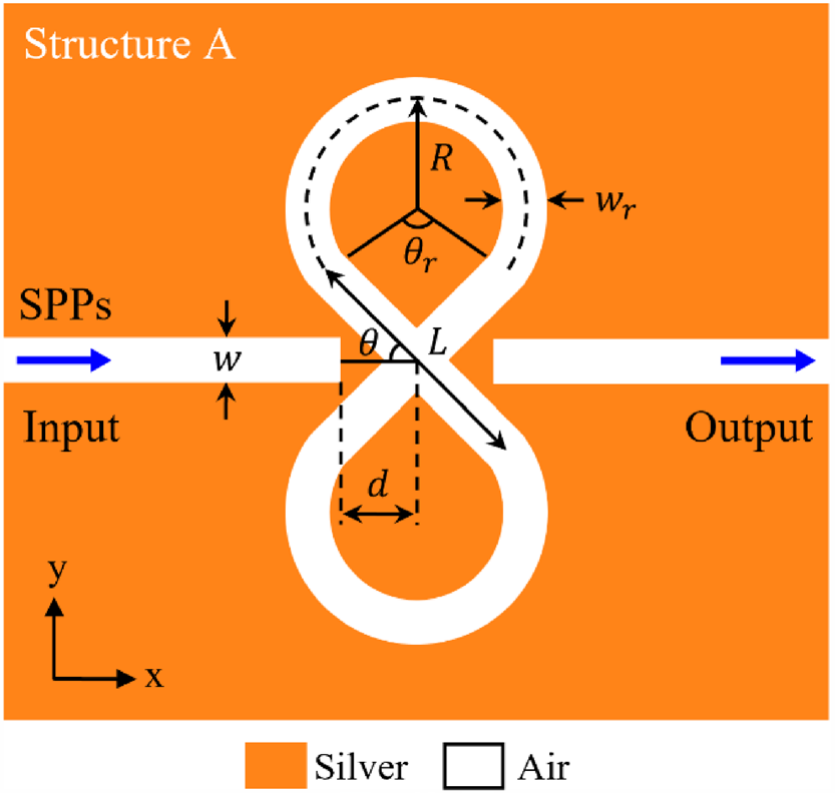
Schematic of the first proposed plasmonic filter (marked as structure A) consisting of two MIM waveguides coupled to each other by an 8-shaped resonator.

Figure 1 shows the structure of the first proposed plasmonic filter (marked as structure A), which consists of two MIM waveguides coupled to each other by an 8-shaped resonator. Here, in the 8-shaped resonator, two cross-passing waveguides are combined with two upper and lower circular rings. The filter’s geometric parameters are the waveguide width ($w$), the width of resonator waveguide (${w}_{r}$), the length of resonator cross-passing waveguides ($L$), the radius of resonator rings ($R$), the angle of resonator rings (${\theta }_{r}$), the distance between the center of the resonator and the lateral MIM waveguides ($d$) and the angle between the resonator branches and the horizontal line ($\theta$). All of the filter’s geometrical parameters are summarized in Table 1. In this plasmonic MIM structure, the insulator or dielectric material is assumed to be air, and the metal is assumed to be silver.

**Table 1:** the geo metrical parameters of the first proposed plasmonic filter (marked as structure A).

Parameter	Symbol	Value
Waveguide widths	$w$	$\text{50 nm}$
Width of resonator waveguide	${w}_{r}$	$\text{50 nm}$
Length of resonator cross-passing waveguides	$L$	$\text{310 nm}$
Radius of resonator rings	$R$	$\text{130 nm}$
The distance between the center of resonator and the lateral MIM waveguides	$d$	$\text{85 nm}$
Angle of resonator rings	${\theta }_{r}$	${110^{\circ}}$
Angle between the resonator branches and the horizontal line	$\theta$	${45^{\circ}}$

Since the width of the input waveguide is much smaller than the incident wavelength ($w\ll \lambda$), only the fundamental transverse magnetic (TM) mode as in the waveguide can exist. For the fundamental TM mode, the dispersion relation of SPPs in the MIM waveguide structures can be expressed as^59^:

1 $${\varepsilon }_{d}{k}_{m}+{\varepsilon }_{m}{k}_{d}\mathrm{tanh}\left(\frac{{wk}_{d}}{2}\right)=0$$

where ${\varepsilon }_{d}$ and ${\varepsilon }_{m}$ are the relative permittivity of the dielectric and metal, respectively. ${k}_{m}$ and ${k}_{d}$ are defined by momentum conservations as follows^59^:

2 $${k}_{m,d}^{2}={\beta }^{2}-{\varepsilon }_{m,d}{k}^{2}$$

where $\beta$ stands the SPP propagation constant. $\beta$ can be calculated using Eqs. (1) and (2). $k=2\pi /\lambda$ is the wave vector in a vacuum. In general, air has a refractive index (n) of 1, while its relative permittivity is ${\varepsilon }_{d}$= 1. The complex relative permittivity of silver is characterized by the well-known Drude model as follows^10^:

3 $${\varepsilon }_{m}\left(\omega \right)={\varepsilon }_{\infty }-\frac{{\omega }_{p}^{2}}{\omega (\omega +j\gamma )}$$

where ${\varepsilon }_{\infty }$ = 3.7 is the dielectric constant at the infinite frequency, ${\omega }_{p}$ = 9.1 eV is the bulk plasma frequency, $\gamma$ = 0.018 eV is the electron collision frequency, $\omega =2\pi c/\lambda$ is the angular frequency of the incident wave, and $c$ is the speed of light in free space.

The effective refractive index (${n}_{\mathrm{eff}}$) of the fundamental TM mode in the MIM waveguide as a function of wavelength $\lambda$ can be solved from ${n}_{\mathrm{eff}}=\beta /k$. The real part of effective refractive index determines the guided wavelength in MIM waveguide (${\lambda }_{\text{SPP}}$) and its imaginary part determines the propagation length of SPPs (${L}_{\text{SPP}}=1/2\mathrm{Im}(\beta )$). Therefore, the effective refractive index (${n}_{\mathrm{eff}}$) can be characterized as follows^60^:

4 $${n}_{\mathrm{eff}}=\frac{\beta }{k}=\left(\frac{\lambda }{{\lambda }_{\text{SPP}}}\right)+j\left(\frac{\lambda }{4\pi {L}_{\text{SPP}}}\right)$$

5 $${n}_{\mathrm{eff}}=\sqrt{{\varepsilon }_{d}}{\left({\varepsilon }_{d}+\frac{\lambda {\varepsilon }_{d}}{w\pi \sqrt{{-\varepsilon }_{m}}}\sqrt{1-\frac{{\varepsilon }_{d}}{{\varepsilon }_{m}}}\right)}^{1/2}$$

**Figure 2:**
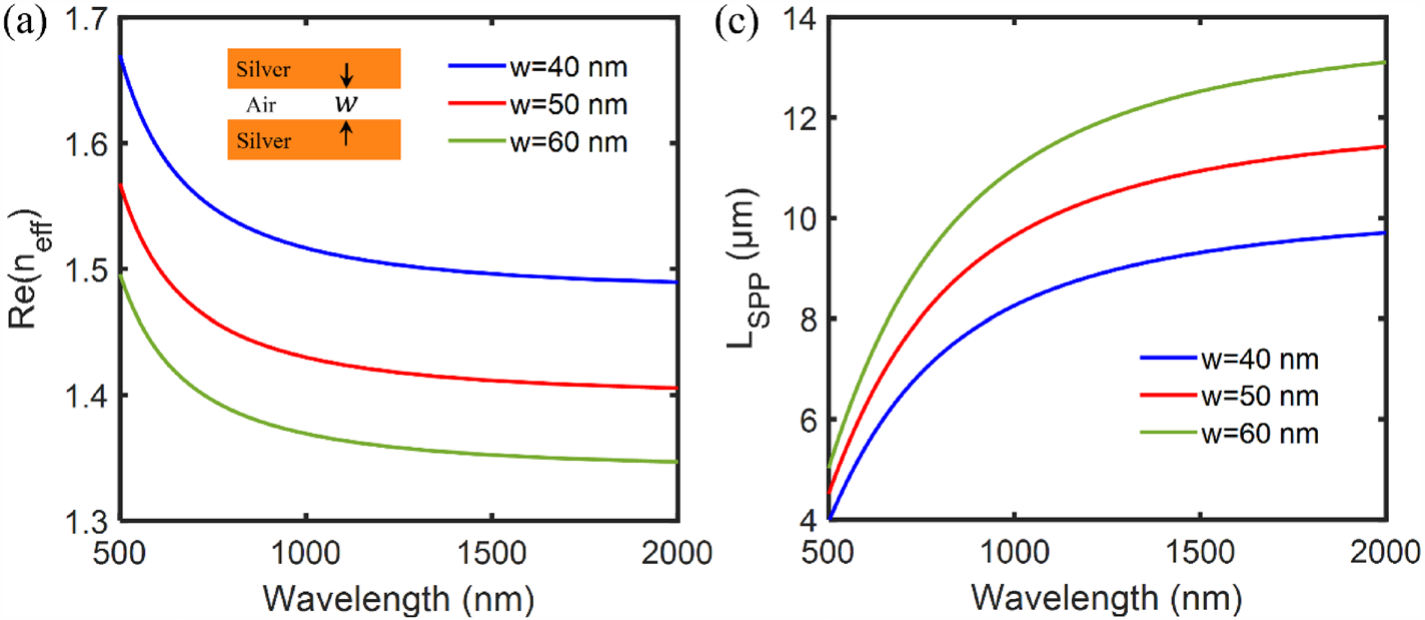
(**a**) Real part of the effective refractive index (${n}_{eff}$) and (**b**) The corresponding propagation length of SPPs (${L}_{\text{SPP}}$) as a function of wavelength for different widths of the air layer in the MIM waveguide.

Based on the above principles, we have calculated the real part of ${n}_{\mathrm{eff}}$ and ${L}_{\text{SPP}}$ for different widths of MIM waveguides $w$ as shown in Fig. 2a,b, respectively. To support only the fundamental TM mode in the MIM waveguide, we set $w=\text{50 nm}$, which is much smaller than the operating wavelengths. Two power monitors are set at input and output ports to detect the incident power ${A}_{1}$ and the transmitted power ${A}_{2}$, respectively. The power transmittance is defined as $T={A}_{2}/{A}_{1}$.

### Ethical approval

We the undersigned declare that the manuscript entitled “A single-mode tunable plasmonic sensor based on an 8-shaped resonator for cancer cell detection” is original, has not been fully or partly published before, and is not currently being considered for publication elsewhere. Also, results are presented clearly, honestly, and without fabrication, falsification, or inappropriate data manipulation. We confirm that the manuscript has been read and approved by all named authors and that there are no other persons who satisfied the criteria for authorship but are not listed. We further confirm that the order of authors listed in the manuscript has been approved by all of us.

## Results and discussion

The finite-difference time-domain (FDTD) method is used to calculate the transmission response of the proposed plasmonic filter. FDTD method is a time-domain solution that solves Maxwell's equations without applying any physical approximation. This method solves Maxwell's equations numerically on a mesh by adopting appropriate boundary conditions. Therefore, the electric and magnetic fields are calculated for all grid points. The perfectly matched layer (PML) boundary conditions are defined at the x- and y-directions of the simulation domain. To propagate SPPs in the waveguide, a mode source as the input light source is launched to the input port of waveguide. Then, a transmission monitor is placed in the output port of waveguide to detect the spectrum transmitted signal. In all directions, the mesh size is set as 2 nm.

**Figure 3:**
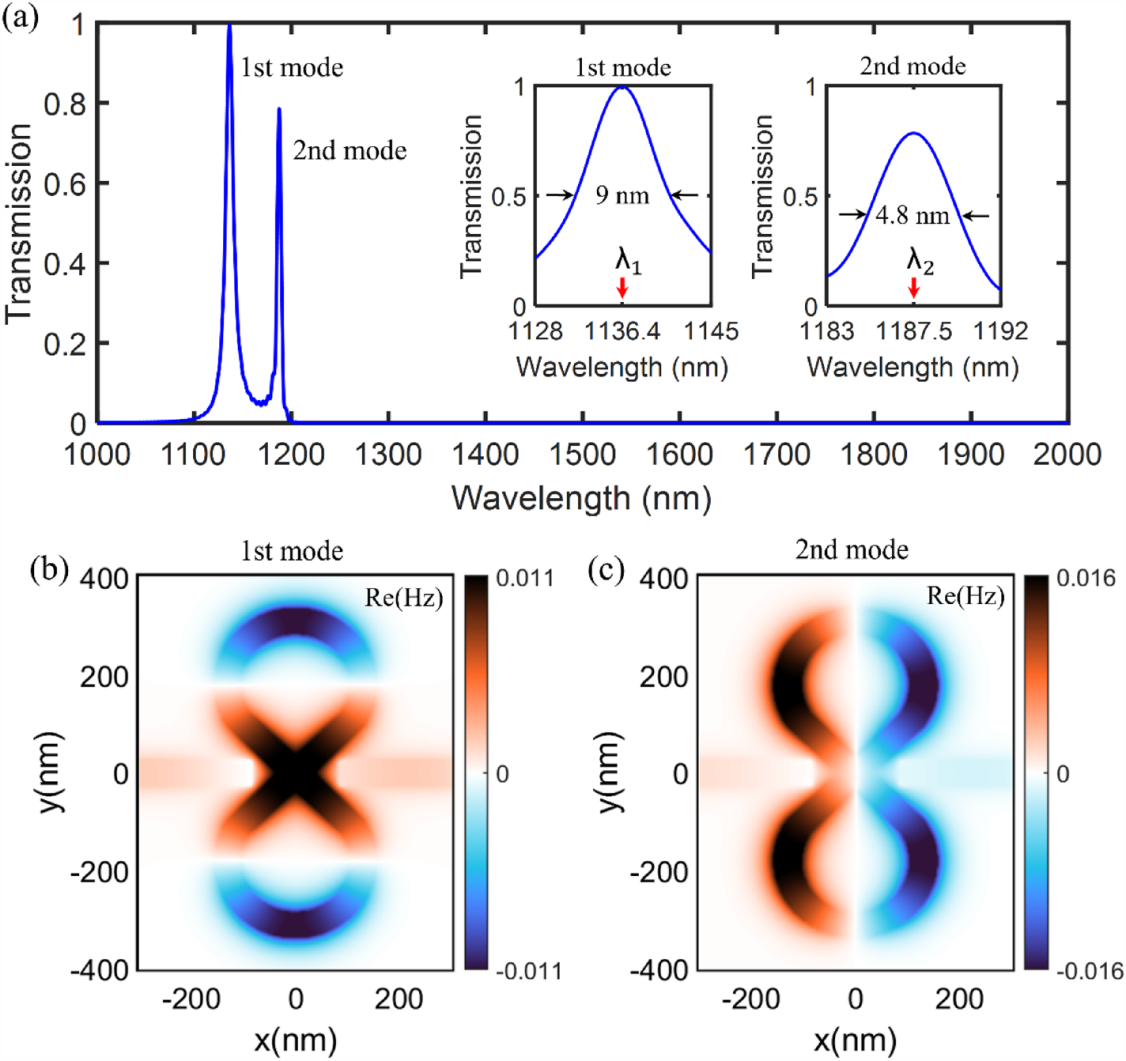
(**a**) The transmission spectrum of the first proposed plasmonic filter (marked as structure A) with an 8-shaped resonator when $w=$ 50 nm, ${w}_{r}=$ 50 nm, $L=$ 310 nm, $R=$ 130 nm, ${\theta }_{r}=$ 110°, $d=$ 85 nm and $\theta =$ 45°. (**b**) The Re(Hz) field pattern of the plasmonic filter at the first resonance wavelength of 1136.4 nm. (**c**) The Re(Hz) field pattern of the plasmonic filter at the second resonance wavelength of 1187.5 nm.

Figure 3a shows the transmission spectrum of the first proposed plasmonic filter with an 8-shaped resonator when $w=\text{50 nm}$, ${w}_{r}=\text{50 nm}$, $L=\text{310 nm}$, $R=\text{130 nm}$, ${\theta }_{r}={110^{\circ}}$, $d=\text{85 nm}$ and $\theta ={45^{\circ}}$. It is observed that there are two resonance modes within the wavelength range of 1000–2000 nm, the first at 1136.4 nm and the second at 1187.5 nm. The first resonance mode has a maximum transmission value of about 100% with an FWHM (full width at half the maximum) bandwidth of about 9 nm. Besides, the second resonance mode has a maximum transmission value of about 80% with an FWHM (full width at half the maximum) bandwidth of about 4.8 nm. Plasmonic filters usually have a broad FWHM bandwidth due to having a larger propagation loss.

To investigate the performance of a resonator, the quality factor (Q-factor) is usually calculated. A lossless or ideal resonator is measured by this parameter. The Q-factor is defined as the ratio of a resonator's resonance wavelength to its bandwidth (${\text{Q}}=\lambda /{\text{FWHM}}$)^61^. In plasmonic resonators, the Q-factor is typically limited by the intrinsic ohmic losses of the metal. Q-factor values of 126.3 and 247.4 have been calculated for the first and second resonance modes.

Figure 3b,c show the electromagnetic field profile in the z-direction (Re(Hz)) for the first proposed plasmonic filter at the 1st and 2nd resonance modes, respectively. As can be seen in the figure, the vivid resonances are created in the 8-shaped resonator. It is evident that the 1st and 2nd resonance modes, respectively originate from the even and odd modes of the electromagnetic field.

To determine the resonance wavelengths in the plasmonic filter, the condition of resonance can be written as^60^:

6 $${L}_{\mathrm{eff}}=N{\lambda }_{\text{SPP}}=m\left(\frac{\lambda }{\mathrm{Re}({n}_{\mathrm{eff}})}\right), N=1, 2, 3, \dots$$

where $N$ is an integer number, which represent the mode numbers. The length of the 8-shaped resonator (${L}_{\mathrm{eff}}$) is calculated as ${L}_{\mathrm{eff}}=2L+\pi R\left(1-{\theta }_{r}/360^\circ \right)$. In this regard, the wavelength associated with each resonance modes will be:

7 $$N \lambda ={L}_{\mathrm{eff}}\mathrm{Re}({n}_{\mathrm{eff}})$$

Using Eqs. (5) and (7), the resonance wavelengths can be calculated as follows:

8 $$\lambda =\frac{{L}_{\mathrm{eff}}}{N}\mathrm{Re}\left[\sqrt{{\varepsilon }_{d}}{\left({\varepsilon }_{d}+\frac{\lambda {\varepsilon }_{d}}{w\pi \sqrt{{-\varepsilon }_{m}}}\sqrt{1-\frac{{\varepsilon }_{d}}{{\varepsilon }_{m}}}\right)}^{1/2}\right]$$

According to Eq. (8), for a specific $N$, increasing the dimensions of the resonator length causes the redshift of resonance wavelengths. Therefore, increasing the length of resonator cross-passing waveguides ($L$) and the radius of resonator rings ($R$) shifts the resonance wavelengths of the filter to higher wavelengths. Hence, the wavelength can be adjusted by changing the parameters of the resonator. It is essential that a high-quality filter be tunable.

**Figure 4:**
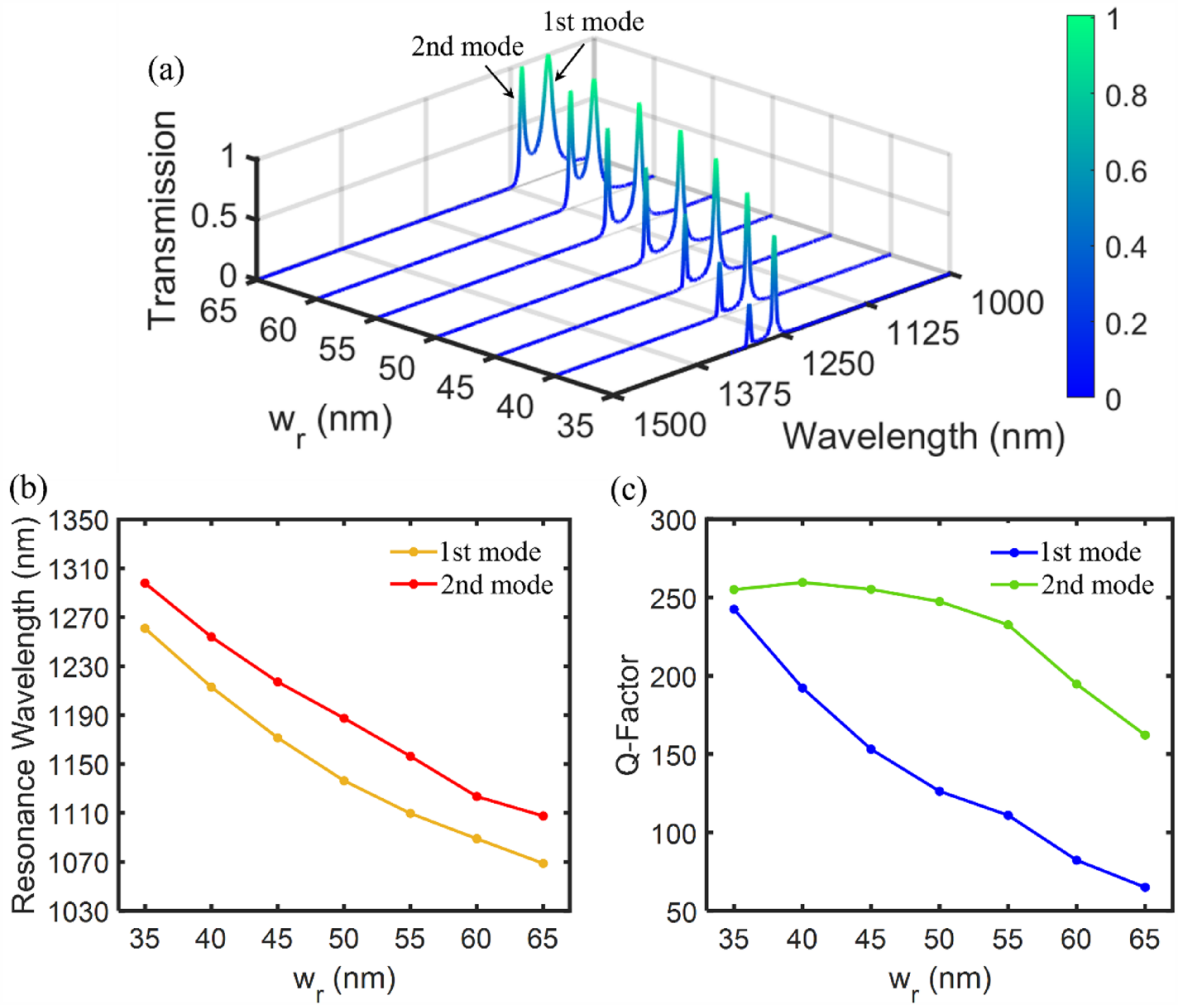
(**a**) The transmission spectrum of the first proposed plasmonic filter (marked as structure A) for different widths of resonator waveguide (${w}_{r}$). (**b**) The first and second resonance wavelengths as a function of the ${w}_{r}$ size. (**c**) The Q-factor of the 1st and 2nd resonance modes as a function of the ${w}_{r}$ size.

**Figure 5:**
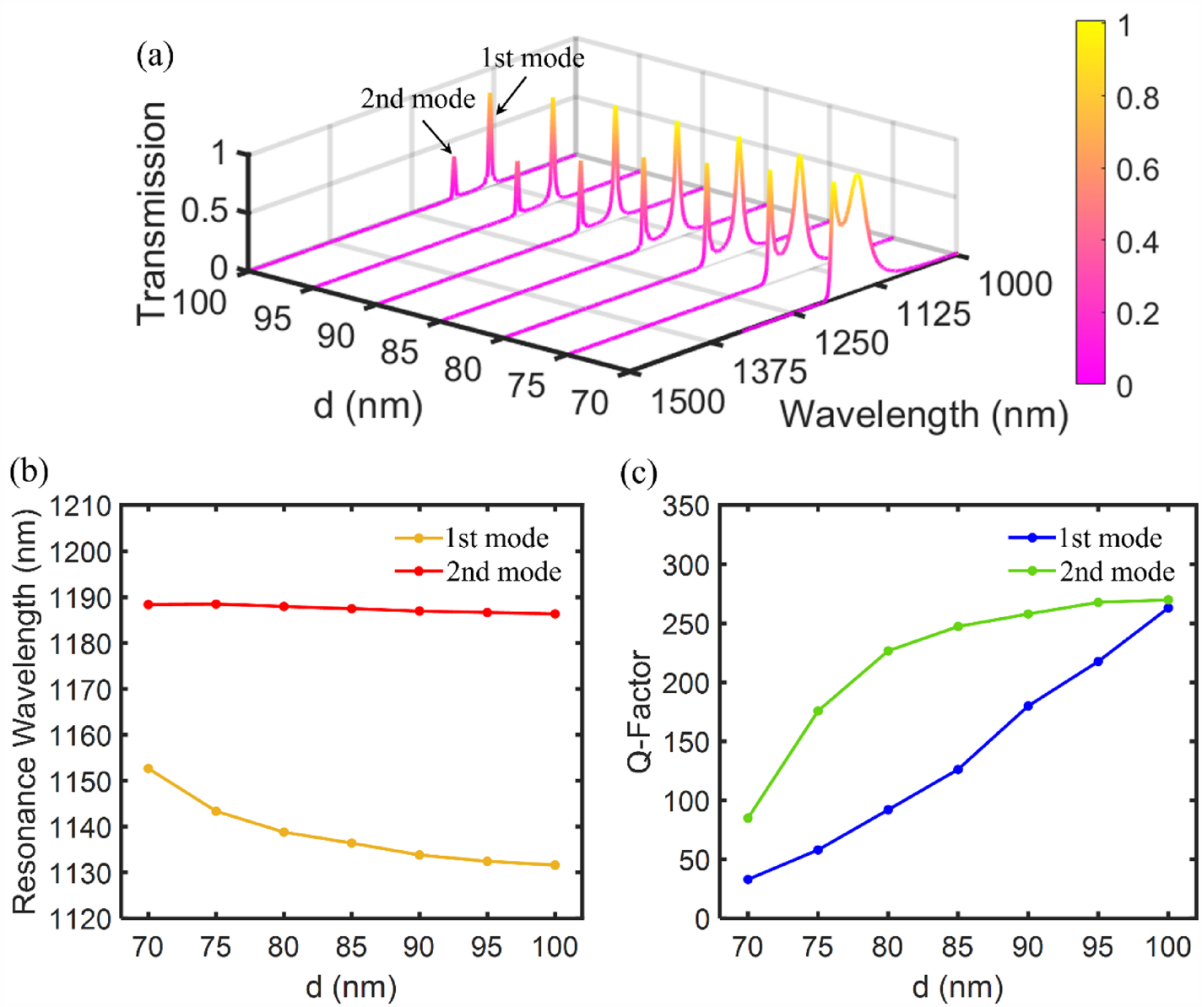
(**a**) The transmission spectrum of the first proposed plasmonic filter (marked as structure A) for different the distances between the center of resonator and the lateral MIM waveguides ($d$). (**b**) The first and second resonance wavelengths as a function of the $d$ size. (**c**) The Q-factor of the 1st and 2nd resonance modes as a function of the $d$ size.

We study the effect of geometric parameters on the transmission spectrum in order to obtain the optimal dimensions of filter structure A. In this regard, we investigate the effects of the width of the resonator waveguide (${w}_{r}$) and the distance between the center of the resonator and the lateral MIM waveguides ($d$) in Figs. 4 and 5, respectively. Figure 4a shows the effect of ${w}_{r}$ on the transmission spectrum of the filter and the corresponding resonance wavelengths. The ${w}_{r}$ size is increased from 35 to 65 nm by a 5 nm step. The other geometric parameters remain unchanged. As shown in Fig. 4b, increasing the ${w}_{r}$ size results in a redshift toward lower wavelengths in the 1st and 2nd resonance modes. The wavelengths of both resonance modes decrease linearly with a slope of about 15.5%. It occurs because the effective refractive index declines with increasing the ${w}_{r}$ size. As a result, according to Eq. (7), the resonance wavelength also decreases. Figure 4c shows the Q-factor of the 1st and 2nd resonance wavelengths of the filter structure A as a function of ${w}_{r}$ size. Increasing the ${w}_{r}$ size results in a sharp reduction of the Q-factor of the first resonance mode from 245 to 65, as well as a decrease of the Q-factor of the second resonance mode from 255 to 160. However, based on Fig. 4a, as the Q-factor is decreased, the transmission peak value is increased when the ${w}_{r}$ size is increased. Consequently, a trade-off is seen between increasing the Q-factor and reducing maximum transmission.

Figure 5a shows the effect of $d$ on the transmission spectrum of filter structure A and the corresponding resonance wavelengths. The $d$ size is increased from 70 to 100 nm in a 5 nm step. The other geometric parameters remain unchanged. As shown in Fig. 5b, as the $d$ size is increased, the 1st and 2nd resonance wavelengths shift toward lower wavelengths. Wavelengths decrease almost linearly in the 1st and 2nd resonance modes with slopes of approximately 0.2% and 2%, respectively. Figure 5c shows the Q-factor of the 1st and 2nd resonance wavelengths of the filter as a function of $d$ size. Increasing the $d$ size results in a sharp increase in the Q-factor of the first and second resonance modes, which reaches a maximum value of about 270 for $d=\text{100 nm}$. Boosting the Q-factor is physically justified according to coupled-mode theory. As the distance between the resonator and the waveguides is increased, the resonance mode will leak less into the waveguides. Therefore, the Q-factor is increased by increasing the $d$ size. However, from Fig. 5a, as the Q-factor is increased, transmission peak value is decreased when the $d$ size is increased. As a result, there is a trade-off between increasing Q-factor and decreasing maximum transmission.

The high-Q plasmonic filter using the 8-shaped resonator (marked as structure A) can be used for sensing applications. However, this structure provides two resonance modes. The overlap of these resonances can complicate spectral identification when the resonances are shifted. Because, two spectra begin to merge and makes monitoring spectral changes difficult. Hence, the second and third structures supporting the single-mode resonance are proposed.

**Figure 6:**
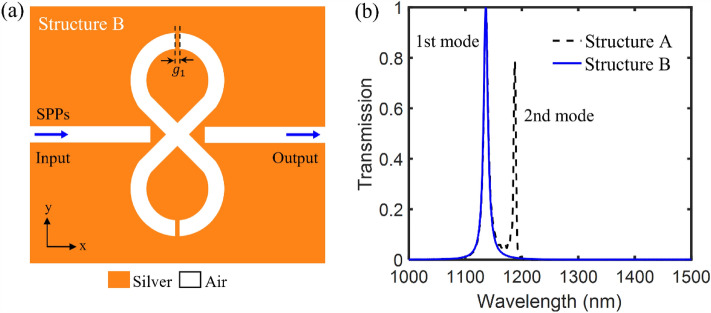
(**a**) Schematic of the second proposed plasmonic filter (marked as Structure B) consisting of the vertical metal gaps in the 8-shaped resonator with a width of ${g}_{1}$. (**b**) The transmission spectrum of the filter structure B supporting a single-mode resonance at the wavelength of 1136.4 nm.

As described previously, the 1st and 2nd resonance modes of the first proposed plasmonic filter (marked as structure A) are derived from the even and odd modes of the electromagnetic field excited in the resonator. To eliminate the odd mode of electromagnetic field, we placed the vertical metal gaps in the 8-shaped resonator. By removing the odd mode, the second resonance mode is eliminated and only the first resonance mode is preserved as a single-mode resonance. Hence, Fig. 6a shows the schematic of the second proposed plasmonic filter (marked as Structure B) consisting of the vertical metal gaps in 8-shaped resonator with a width of ${g}_{1}$. Figure 6b shows the comparison of the transmission spectrum of the plasmonic filter structures A and B. For filter structure B, the width of metal gap is set to be ${g}_{1}=\text{5 nm}$, while other geometric parameters are based on Table 1. For filter structure B, it can be seen that the 2nd resonance mode is removed and only the 1st resonance mode is preserved. Therefore, the second proposed plasmonic filter provides a tunable single-mode resonance at a wavelength of 1136.4 nm.

**Figure 7:**
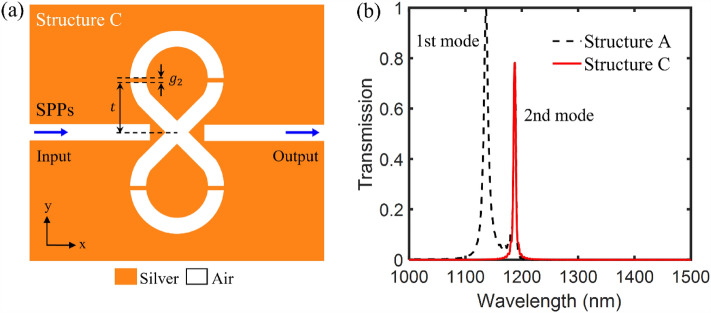
(**a**) Schematic of the third proposed plasmonic filter (marked as Structure C) consisting of the horizontal metal gaps in the 8-shaped resonator with a width of ${g}_{2}$. (**b**) The transmission spectrum of the filter structure C supporting a single-mode resonance at the wavelength of 1187.5 nm.

Furthermore, to eliminate the even mode of electromagnetic field, we placed two horizontal metal gaps in the 8-shaped resonator. By removing the even mode, the 1st resonance mode is eliminated and only the 2nd resonance mode is preserved as a single-mode resonance. In this regard, Fig. 7a shows the schematic of the third proposed plasmonic filter (marked as Structure C) consisting of the horizontal metal gaps in the 8-shaped resonator with a width of ${g}_{2}$. Figure 7b shows the comparison of the transmission spectrum of the plasmonic filter structures A and C. For filter structure C, the width of metal gap is set to be ${g}_{2}=\text{5 nm}$, and the distance between the center of the resonator and the horizontal metal gap is $t={142}\text{.5 nm}$. Other geometric parameters are based on Table 1. For filter structure C, it can be seen that the fist resonance mode is removed and only the second resonance mode is preserved. Therefore, the third proposed plasmonic filter provides a tunable single-mode resonance at the wavelength of 1187.5 nm.

**Figure 8:**
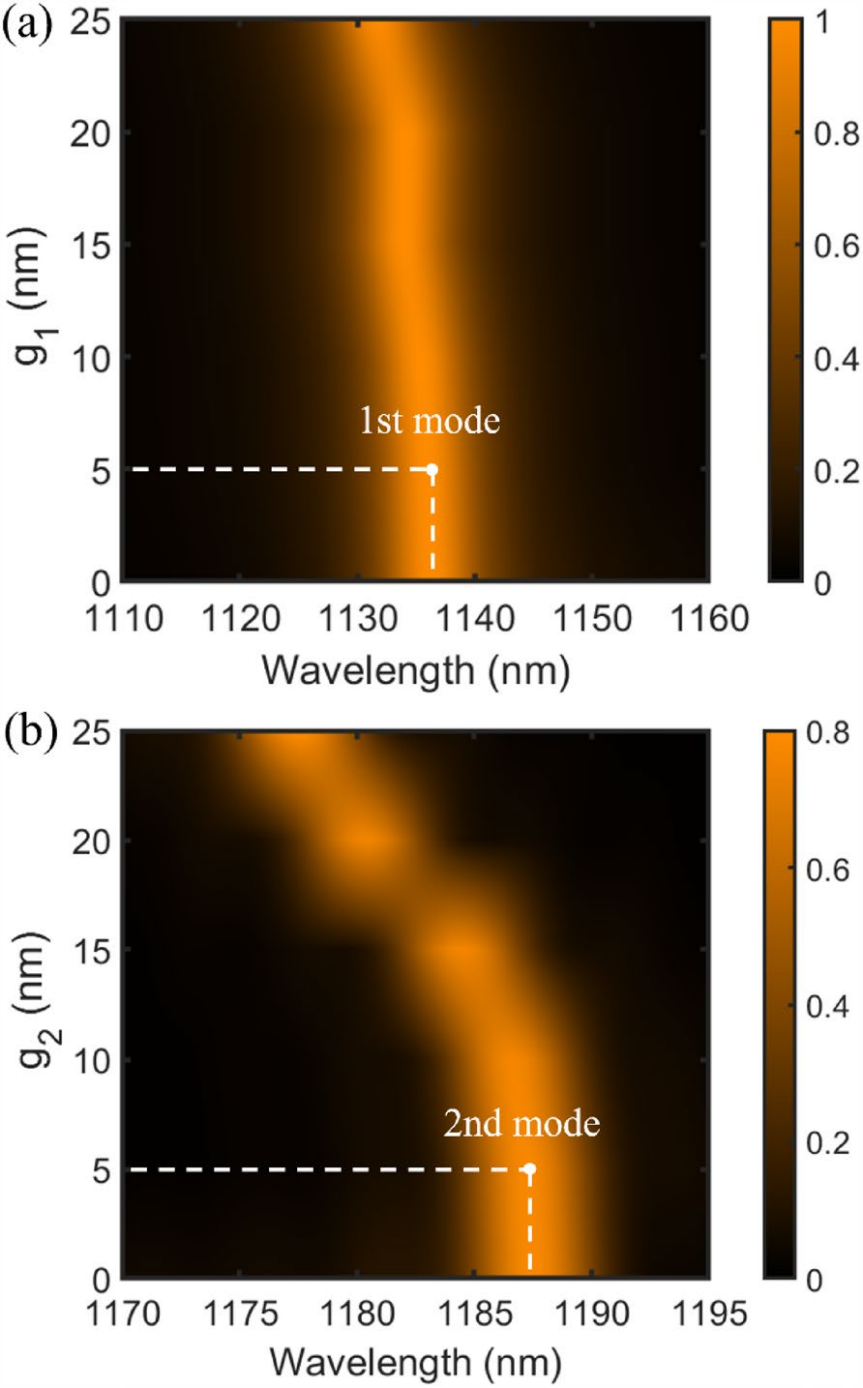
(**a**) The transmission spectrum of the second proposed plasmonic filter (marked as structure B) for different widths of vertical metal gap (${g}_{1}$). (**b**) The transmission spectrum of the third proposed plasmonic filter (marked as structure C) for different width of horizontal metal gap (${g}_{2}$).

Figure 8a shows the effect of varying ${g}_{1}$ on the transmission spectrum of filter structure B and the corresponding 1st resonance single-mode wavelength. The ${g}_{1}$ size is increased from 0 to 25 nm in a 5 nm step. The other geometric parameters of filter structure B are fixed. As seen, by incrementing ${g}_{1}$ size, the 1st single-mode wavelength shifts toward lower wavelengths. Single-mode resonance at a wavelength of 1136.4 nm can be achieved for ${g}_{1}=\text{5 nm}$. Moreover, Fig. 8b shows the effect of varying ${g}_{2}$ on the transmission spectrum of filter structure C and the corresponding 2nd resonance single-mode wavelength. The ${g}_{2}$ size is increased from 0 to 25 nm in a 5 nm step. The other geometric parameters of filter structure C are fixed. As seen, increasing ${g}_{2}$ size results in the shift toward lower wavelengths for the 2nd single-mode wavelength. Therefore, single-mode resonance at a wavelength of 1187.5 nm can be achieved for ${g}_{2}=\text{5 nm}$.

## Biosensing application

**Figure 9:**
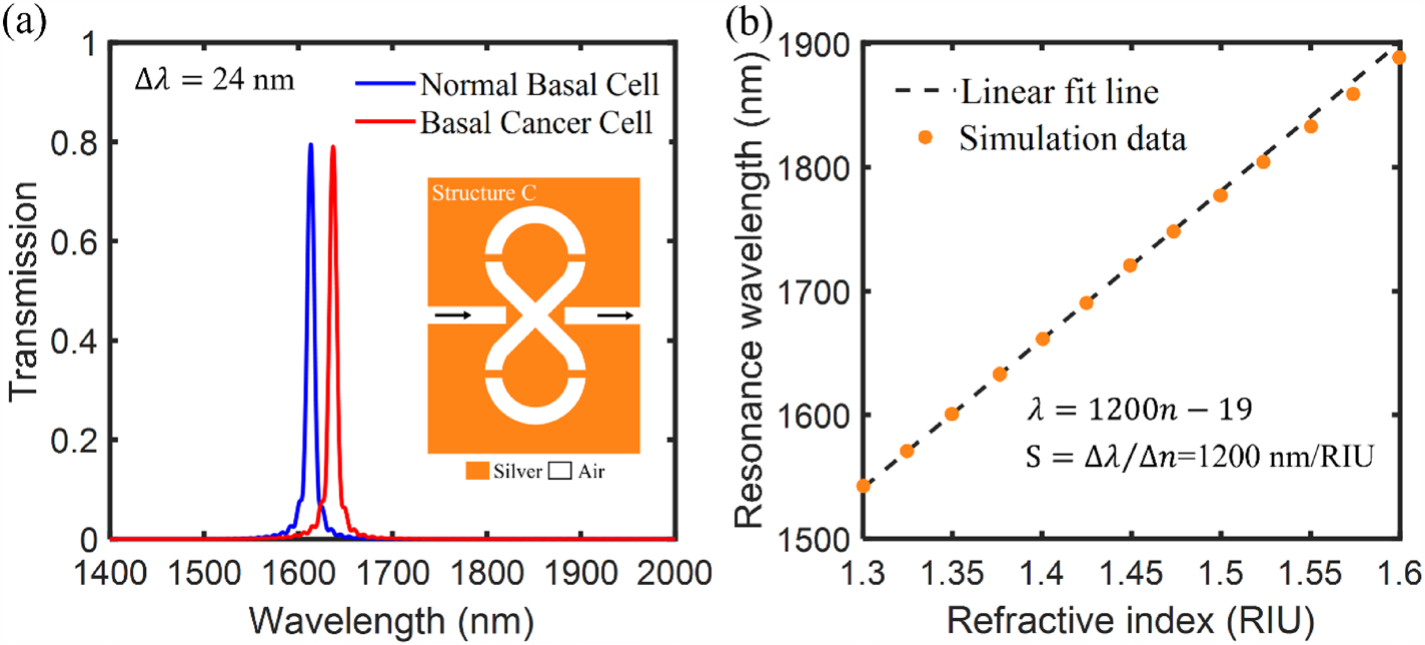
(**a**) The transmission spectrum of third proposed plasmonic filter (marked as structure C) corresponding to the normal basal cell and basal cancer cell. (**b**) Resonance wavelength of the proposed third structure as a function of environment refractive indices.

In this section, it is shown that third proposed tunable plasmonic filter structure can be used for biosensing applications such as the detection of basal cancer cell. Basal cancerous cell is responsible for skin cancer in human body. The cancerous tumor has a refractive index of 1.38, while the normal basal cell has a refractive index of 1.36^62^. The change in the refractive index from normal to cancer cell is very small. To detect this change the sensor design must be highly sensitive. Since the refractive indices of human cells are measured in the near infrared range, the proposed tunable filter structure can be used for sensing normal and cancerous cells. Figure 9a shows the transmission spectrum of third proposed plasmonic filter corresponding to the normal basal cell and basal cancer cell. The refractive index variation occurs in the surrounding environment of structure after proximity to the cells. Consequently, changing the refractive index shifts the resonance wavelength in the transmission spectrum of the sensor structure. As seen, the resonance wavelength shift in the transmission spectrum of third proposed structure is 24 nm. To investigate the sensing performance of proposed biosensor structure, the sensitivity (S) parameter is calculated. The sensitivity (S) of sensor is defined as the ratio of the resonance wavelength shift to the refractive index variation (${\text{S}}=\Delta \lambda /\Delta n$)^63^. Figure 9b shows a linear relationship between the resonance wavelength and the environment refractive index variations from n = 1.3 to 1.6 in the near infrared range. As can be seen, the calculated slope of the linear fit line estimates the sensitivity of sensor. Hence, the sensitivity value of 1200 nm/RIU is obtained. Therefore, the third proposed plasmonic filter structure as a plasmonic biosensor can be a good candidate for the detection of basal cancer cell.

To provide a better view of the obtained results in this paper, the third proposed plasmonic structure (marked as structure C) is compared with other reported works in recent years. For comparison, Table 2 shows some main characteristics of refractive index plasmonic sensors based on MIM waveguides. The comparison parameters include the filter shapes, topologies, Detection, resonance wavelength ($\uplambda$), Q-factor, and sensitivity. Our proposed plasmonic structure is a tunable filter that exhibits single-mode behavior in the 1000–2000 nm range. The resonance wavelength can be adjusted by changing the structural parameters of the resonator. The proposed tunable filter has a Q-factor of 247.4, which is higher than most of the works presented in Table 2. Moreover, this structure can be used as a plasmonic biosensor for the detection of basal cancer cells. It depicts the highest sensitivity of 1200 (nm/RIU) among the many published sensor structures in this table. In most plasmonic sensors, the sensitivity and Q-factor are not simultaneously improved. It can be seen, our proposed structure is clearly superior both in terms of sensitivity as well as Q-factor to most recent sensors reported.

**Table 2:** Comparison of the sensing performance of the proposed plasmonic biosensor with other works.

References	Year	Setup	Topology	Detection application	$\uplambda$(nm)	Q-factor	Sensitivity (nm/RIU)
^4^	2016	MIM	Double rectangular cavities	General	620	–	596
^64^	2017	MIM	Rectangular side-coupled with a disk cavity	General	1148.5	85.77	1136
^65^	2017	MIM	Two silver baffles and a coupled ring cavity	General	710	–	718
^66^	2018	MIM	Pair of elliptical ring resonators	Hemoglobin	≃1004	200.8	1100
^67^	2019	MIM	Si nano-ring located inside a circular cavity	General	808	269.3	636
^68^	2019	MIM	Tooth cavity-coupled ring splitting cavity	General	1055	107.32	1200
^69^	2019	MIM	Side-coupled stub-hexagon resonators	General	915	140.122	937.5
^70^	2020	MIM	Two rectangular nanocavities coupled to a Bus waveguide and two nanodisk resonators	General	796	21.84	750
^31^	2020	MIM	Three quadrilateral cavities sandwiched perpendicularly in between the MIM waveguides	Blood group	1651	≃ 15.7	1556
^71^	2021	MIM	An asymmetric cross-shaped resonator	General	839	–	795
^72^	2021	MIM	Two sequential ring resonators	General	998.8	132.8	1000
^73^	2021	MIM	Three H-shaped cavities	General	1056.4	69.91	1050
^74^	2022	MIM	Split-ring Heptamer structures	General	1600	–	840
This work (Structure C)	2023	MIM	8-shaped resonator	Basal cancer cells	1187.5	247.4	1200

## Conclusions

In this paper, a tunable plasmonic filter, which consists of two MIM waveguides coupled to each other by an 8-shaped resonator was designed and investigated. This structure had two resonance modes at wavelengths of 1136.4 nm and 1187.5 nm in the 1000–2000 nm range. The calculated Q-factor for resonance modes was obtained equal to 126.3 and 247.4, respectively. The performance of the structure was also theoretically analyzed. Then, by placing metal gaps in the vertical and horizontal directions of the resonator, two tunable single-mode plasmonic filters, to support each of the former resonance modes, were proposed. Finally, the proposed structure with the horizontal metal gaps was investigated for sensing applications. The effect of structural parameters on the transmission spectrum and the sensing performance of the sensor were investigated using the finite-difference time-domain (FDTD) method. Based on the obtained results, this structure can be used to detect the basal cancer cells with a sensitivity of 1200 nm/RIU. The main drawback of many plasmonic sensors is that both sensitivity and Q-factor are not improved simultaneously. While for our proposed structure, they are much better than the many recently reported sensors. Accordingly, the work presented in this paper is a suitable candidate for filtering and sensing applications in optic communications.

## Data Availability

The datasets generated and analyzed during the current study are available from the corresponding author on reasonable request.
